# Evaluation of the effectiveness and cost-effectiveness of the chronic disease co-care (CDCC) Pilot Scheme: a study protocol

**DOI:** 10.1186/s12875-025-02765-6

**Published:** 2025-03-19

**Authors:** Ivy L. Mak, Kiki S.N. Liu, Zoey C.T. Wong, Vivian Y.H. Xu, Esther Y.T. Yu, Tony K.H. Ha, William C.W. Wong, Emily T.Y. Tse, Linda Chan, Amy P.P. Ng, Edmond P.H. Choi, Martin Roland, David Bishai, Cindy L.K. Lam, Eric Y.F. Wan

**Affiliations:** 1 Department of Family Medicine and Primary Care, Li Ka Shing Faculty of Medicine, The University of Hong Kong, Hong Kong, China; 2 Primary Healthcare Commission, Health Bureau, Hong Kong, China; 3 Bau Institute of Medical and Health Sciences Education, Li Ka Shing Faculty of Medicine, The University of Hong Kong, Hong Kong, China; 4 School of Nursing, LKS Faculty of Medicine, The University of Hong Kong, Hong Kong, China; 5 Department of Public Health and Primary Care, The University of Cambridge, Cambridge, England; 6 School of Public Health, Li Ka Shing Faculty of Medicine, The University of Hong Kong, Hong Kong, China; 7 Centre for Safe Medication Practice and Research, Department of Pharmacology and Pharmacy, LKS Faculty of Medicine, The University of Hong Kong, Hong Kong, China; 8 The Institute of Cardiovascular Science and Medicine, Faculty of Medicine, The University of Hong Kong, Hong Kong, China; 9 Advanced Data Analytics for Medical Science, Hong Kong, China; 10 Department of Family Medicine and Primary Care, The University of Hong Kong-Shenzhen Hospital, Shenzhen, China

**Keywords:** Diabetes mellitus, Hypertension, Screening, Management, Multi-disciplinary, Primary healthcare, Quality of care, Effectiveness, Cost-effectiveness

## Abstract

**Background:**

The Chronic Disease Co-Care (CDCC) Pilot Scheme is a government-subsidized program that aims to provide targeted copayment for the screening and management of hypertension, diabetes mellitus and pre-diabetes in the private healthcare sector. Studies have found that concurrent screening and management with a multi-disciplinary intervention is cost-saving because of the reduction in the rates of premature mortality, complications and utilization of public health services. This study aims to evaluate the quality of care, acceptability, effectiveness and cost-effectiveness of the CDCC Pilot Scheme.

**Methods:**

Quality of care will be evaluated by the standards achieved by the program in each criterion in the domains of structure, process and outcomes of care. Site visits and two serial questionnaire surveys at 6 and 12 months will be conducted for the structure of care. Operational data, including the provision of diagnosis and treatment, as well as participants’ health status will be extracted to evaluate the process and outcomes of care. Participants’ acceptability will be evaluated on experience (accessibility, facility, continuity of care and communication), satisfaction (perceived usefulness, continuation and recommendation) and enablement in 548 CDCC participants at 3 and 12 months by telephone surveys. Evaluation of the effectiveness and cost-effectiveness is a 1-year comparative cohort study using longitudinal data on changes in disease control parameters between CDCC and non-CDCC participants at baseline and 12 months. Costing questionnaires on the set-up and operation costs of the Scheme among service providers, and direct medical costs incurred from public and private service utilization among participants within 12 months from enrolment will be assessed. The incremental costs incurred for an additional participant in the CDCC Pilot Scheme to achieve target disease control outcomes after 12 months will be reported as an indicator for cost-effectiveness.

**Discussion:**

The quality of care and effectiveness of the CDCC Pilot Scheme in enhancing the health outcomes of the Scheme participants will be examined. Standards of good practice and recommendations for quality enhancement will be established to inform service planning in similar cross-sector screening and management programme.

**Trial registration:**

NCT06310148; 2024-03-22.

**Supplementary Information:**

The online version contains supplementary material available at 10.1186/s12875-025-02765-6.

## Background

The Hong Kong public healthcare system is faced with ever-increasing challenges brought about by an aging population, greater prevalence of chronic diseases, rising costs of medical care, as well as increased citizen expectations from health systems. Reconfiguration of the primary healthcare (PHC) system to a preventive-oriented system equipped with multi-disciplinary teams could enable healthcare systems to be more effective and efficient. There is compelling international evidence suggesting that having a family doctor (FD) as a regular source of primary care is associated with significantly lower odds of avoidable hospitalization in chronic disease patients [1–3], as well as improved patient outcomes [4, 5].

To alleviate the growing burden placed by chronic diseases on the public healthcare system, the Hong Kong government has developed the Primary Healthcare Blueprint outlining strategies to strengthen district-based primary care services across Hong Kong. Among these strategies include the early identification and intervention of chronic diseases through disease screening at the community level, streamlining the referral system, and engaging the private healthcare sector to improve accessibility to quality PHC.

Specifically, the Chronic Disease Co-Care (CDCC) Pilot Scheme was launched in November 2023 to provide targeted subsidy for the screening and management of hypertension (HT), diabetes mellitus (DM) and pre-diabetes (pre-DM) in the private healthcare sector. Studies have found that screening adults for DM and pre-DM is cost-saving from a health system’s perspective and can lower pre-mature mortality rates [6, 7]. In addition, concurrent screening and management of HT and DM with a multi-disciplinary intervention has been shown to be cost-saving in part because of the reduction in complications and subsequent utilization of public secondary and tertiary health services [8, 9].

Participants of the CDCC Pilot Scheme will receive protocol-driven multi-disciplinary risk assessment and management in private PHC allowing for timely intervention to prevent complications of HT/DM. To ensure the quality of the provision of continuous and holistic primary care, Scheme FDs must be registered in the Primary Care Directory and use the PHC reference frameworks as the standard care protocol for PHC services. District Health Centres (DHC) and DHC Express throughout the territory will serve as case managers to support FDs and coordinate with healthcare service providers to deliver network allied health services, laboratory service and drug service. According to the diagnosis, FDs will recommend comprehensive health management plans, provide necessary medication treatment, and set health targets together with participants. To support participants in disease prevention and management, nurse-led clinic will provide education, health assessment, and counselling services; whereas allied health professionals (including optometrists, podiatrists, dietitians, and physiotherapists) will provide individualised intervention services for participants. Each Scheme participant will be entitled to co-payment for services received under the CDCC Pilot Scheme which include subsidised FD, allied health, drug, and laboratory investigation services.

In order to assure that the CDCC Pilot Scheme is delivered with the intended care and achieves the expected health benefits, we will evaluate its quality of care, acceptability, effectiveness and cost-effectiveness to inform future PHC planning and policy.

## Aims and objectives

This study aims to evaluate the quality of care, acceptability, effectiveness and cost-effectiveness of the CDCC Pilot Scheme. The objectives of this study are to:

- Measure the adherence to the standards of care criteria by healthcare providers participating in the Scheme;
- Evaluate participant acceptability by self-reported outcomes on enablement, experience and satisfaction level about the services of the Scheme;
- Evaluate the effectiveness of the Scheme in improving health outcomes among CDCC participants over 12 months; and.
- Determine the provider costs and cost-effectiveness for delivering the CDCC Pilot Scheme relative to its effectiveness on participants health outcomes.

We hypothesize that:

- Healthcare providers participating in the Scheme would achieve over 70% adherence for the standards of care criteria;
- 60% of participants will report an increase in patient enablement levels after 12 months;
- 90% of participants will report patient satisfaction 12 months after enrolment;
- A greater proportion of participants in the CDCC Pilot Scheme would achieve targets for disease control outcomes after 12 months compared to the comparison group; and.
- The Scheme would be cost-effective based on an incremental cost-effectiveness ratio (ICER) lower than the willingness-to-pay threshold of 1 annual gross domestic product (GDP) per person.

## Methods

### Study design

This study consists of three parts. First, the evaluation of the quality of care is a cross-sectional study including site visits at DHC/DHC Express, administration of two serial quality of care questionnaires at 6 and 12 months among service providers, and extraction of the Scheme operational data from the eHealth database and DHC IT module, which are electronic databases used by the DHC/DHC Express to record participant data. Second, the evaluation of participant acceptability is a longitudinal cohort study by telephone surveys among CDCC participants at 3 and 12 months after CDCC enrolment. Third, the evaluation of the effectiveness and cost-effectiveness is a 1-year comparative cohort study using longitudinal data on health outcomes and healthcare utilization (and hence direct medical costs) among CDCC and non-CDCC participants at baseline and 12 months. Costing questionnaires on the set-up and operation costs of the Scheme will be distributed among service providers.

### Subjects

To evaluate the quality of care, all operators of the DHC/DHC Express, and selected FDs and allied health professionals, will be asked to complete quality of care questionnaires. All participants who have enrolled into the CDCC Pilot Scheme between 1 Jan and 30 Jun 2024 will be included in the evaluation of the standards on process and outcome of care.

A convenient sample of 548 CDCC participants would be recruited from all 18 DHC/DHC Express between 1 Apr 2024 and 31 Aug 2024 to complete a survey on self-reported outcomes on participant acceptability and enablement. A conservative target of 140 participants for each diagnosis (i.e., normal, pre-DM, HT and DM) will be set to ensure different patients are equally represented.

The effectiveness and cost-effectiveness of the Scheme will be evaluated by comparing the differences in health outcomes and healthcare utilization rates among CDCC and non-CDCC participants at 12 months after enrolment. Records for CDCC participants enrolled between 1 Jan and 30 Apr 2024 who meet the inclusion criteria to participate in the CDCC Pilot Scheme will be included as the intervention group, while participants in the comparison group would be non-CDCC participants recruited in the community through collaboration with local networks and organizations. Participants ≥ 45 years, without a known diagnosis of HT and/or DM, and provide informed consent are eligible to join the study.

### Sample size calculation

The sample size needed for evaluating participant acceptability is based on the proportion of participants who showed an increase in enablement, measured by the Patient Enablement Instrument (PEI), one year after enrolment into the CDCC Pilot Scheme. Previous studies showed that 55.6–72.2% of participants reported PEI score > 0 after entering structured programmes for DM and/or HT [10–12]. Assuming the proportion of CDCC participants with PEI score > 0 at 12 months to be 50%, 383 CDCC participants are required with 5% margin of error and significance level of 5%. To allow for about 30% attrition, the sample size required will be 548.

The sample size for evaluating the effectiveness and cost-effectiveness was determined based on the difference in the proportion of patients achieving the targeted outcome for disease control after 12 months between CDCC participants and the comparison group. Previous studies evaluating the 12-month effectiveness of the multi-disciplinary Risk Assessment and Management Program for DM or HT implemented in public primary care in Hong Kong found that the proportion of participants achieving treatment target (glycated hemoglobin [HbA1c] < 7.0% or systolic/diastolic blood pressure [BP] < 140/90mmHg) increased by 5.40% [13] or 1.18% [14], respectively after 12 months, compared to patients receiving usual care. Assuming a conservative 3% increase in the proportion of participants achieving treatment target after 12 months in the CDCC group, a total of 378 participants with pre-DM, HT and/or DM are required to detect difference at a power of 80% with a significance level of 5%/ According to the Hong Kong Population Health Survey 2020, the prevalence of undiagnosed HT and DM among individuals aged 45–84 was 15.4% and 4.6%, respectively [15]. Previous studies reported that the prevalence of pre-DM was 2.3–8.9% in Hong Kong [15, 16]. Taking a prevalence of approximately 20% in total for pre-DM, HT and/or DM, and accounting for an attrition of 30%, a total of 1,886 non CDCC participants will be required.

### Data collection

#### Evaluation of the quality of care

The CDCC Pilot Scheme will be evaluated based on a systematic and user-centric approach described by Donabedian et al. [17], where components of the programme are classified into a taxonomy of structure, process and outcome of quality of care. Structure refers to the conditions which care is provided including infrastructure (facilities and equipment), human resources, staff training and organizational characteristics. Process refers to the set of activities that go on within and between healthcare providers and patients, i.e., the provision of diagnosis, treatment, prevention, and education etc. Outcomes refer to the changes in patients’ health that can be attributed to healthcare, and may include changes in health status, patient knowledge, behavior and patient satisfaction.

Evidence-based quality of care indicators and standards on the structure, processes and outcomes will be established based on the operation protocols developed by the Primary Healthcare Commission, and in consultation with the service providers. The evaluation framework is attached in Appendix A. Site visits at DHC/DHC Express by the research team were carried out between June and August 2024 to understand the programme operation. A site visit form with a standardized flow and questions asked during the site visit was developed to ensure consistency in data collection. Data on the structure of care are collected through questionnaires completed by the operation managers at DHC/DHC Express at 6 and 12 months, and by a representative sample of FDs and allied health professionals at 12 months. Data on the process and outcomes of care will be extracted from eHealth and DHC IT module. Compliance to standards of care across different service providers will be measured based on the evaluation framework.

#### Evaluation of participant acceptability

Questionnaires will be administered via an online or telephone survey at 3 and 12 months following their enrolment into the Scheme by an independent professional survey provider. The surveys will measure participant experience, satisfaction and enablement with the early (i.e. screening and FD pairing) and late (i.e. treatment and management) stages of the CDCC Pilot Scheme.

The questionnaire to evaluate participant experience and satisfaction was adapted based on existing validated instruments used in evaluation studies on general healthcare services and chronic disease management [18–21], including one that has been validated in the Hong Kong population [22]. Participant experience will be evaluated on four domains including accessibility, facility, continuity of care and communication. Accessibility covers the concerns of location, finance, appointment time and range of choices for appointment times. Facility refers to the logistics of service such as opening hours and service duration. Continuity of care is indicated by visiting the same doctor and coordination of multi-disciplinary healthcare. Communication consists of a clear explanation, management plan, manner and participants’ confidence. Satisfaction is subcategorized into perceived usefulness, continuation and recommendation of the service and programme. There are three types of responses, including dichotomous response (e.g. yes/no), 4-point Likert scale rating (e.g. 1 = strongly disagree/never; 2 = agree/rarely; 3 = disagree/sometimes; 4 = strongly disagree/always), and open-ended question (e.g. waiting/consultation time). The questionnaire is attached in Appendix B.

The PEI will be used to quantify participant enablement in coping with their own health status [23]. It has been widely used in the primary care setting [11, 24] and the traditional Chinese version has shown to be valid and reliable among the Chinese population [25]. It consists of 6 items rated using a 3-point scale (0 = the same or less; 1 = slightly improved; 2 = greatly improved) with the summated score ranging from 0 to 12. A higher PEI score indicates better patient enablement.

#### Evaluation of the effectiveness and cost-effectiveness

Anonymous data including demographics, diagnoses, prescriptions and laboratory tests (e.g. full lipid profile and renal function test) at baseline and 12 months for CDCC participants will be extracted from the eHealth database and DHC IT module, whereas those for non-CDCC participants will be collected via a telephone survey by an independent professional survey provider, and they will complete the screening blood test at a designated laboratory. With the exception of critical results that requires immediate medical attention, participants of the comparison group will be asked to follow up on any abnormal results on their own.

Costs of the CDCC Pilot Scheme composes the of implementation and operation costs by health service providers and the direct medical costs of participants. The total costs of implementing CDCC services will include all expenditure incurred from setting up the programme until 31 Dec 2024, while ongoing operational costs would be collected over a 1-year period from Dec 2023– Dec 2024 (Appendix C). Costs will be collected from each DHC/DHC Express, a representative sample of service providers and the Primary Healthcare Commission at 12 months using a costing questionnaire.

Direct medical costs refer to the ongoing costs on utilization of public and private healthcare services (including hospitalization, Accident & Emergency department, General Out-patient Clinics, and Specialist Out-patient Clinics) for both CDCC and non-CDCC participants, as well as the co-payment for the CDCC services among CDCC participants, within 12 months from enrolment. The information will be extracted anonymously from the Hospital Authority’s eHealth database and Clinical Management System, and collected from participants through telephone survey at 12 months (Appendix D). The cost of public healthcare service utilization will be estimated by multiplying the frequency of utilization with the unit cost published in the Gazette and Hospital Authority ordinance (Chap. 113) of charges for non-entitled persons for each public healthcare service.

### Outcome measures

#### Primary

1. The proportion of participants and service providers achieving each criterion of the evaluation framework.
2. Participant enablement measured by the PEI at 3 and 12 months.
3. Differences in the proportion of CDCC and non-CDCC participants achieving the targeted disease control outcome after 12 months (systolic/diastolic BP < 140/90mmHg for HT, reduced HbA1c for pre-DM, HbA1c < 7% for DM).
4. Differences in health service utilization rate and direct medical costs of CDCC and non-CDCC participants within 12 months from enrolment.
5. The ICER for achieving the targeted outcomes for disease control.

#### Secondary

1. Participant experience and satisfaction of the CDCC services at 3 and 12 months.
2. Differences in the changes in mean BP or HbA1c between CDCC and non-CDCC participants with HT, pre-DM and/or DM from baseline to 12 months.
3. Costs for the implementation and operation of the CDCC Pilot Scheme in its pilot phase (1–2 years).

### Data analysis

The proportion of patients and/or service providers achieving the standards of care, participant experience, satisfaction and enablement, and the costs of the CDCC Pilot Scheme and the direct medical costs from the participants at 12 months will be described with descriptive statistics. Differences in the proportion of participants reaching the targeted disease control outcomes between CDCC and non-CDCC participants will be compared using chi-squared tests, and adjusted for confounding factors by logistic regression. Changes in clinical parameters from baseline to 12 months will be compared between CDCC and non-CDCC participants by paired t-tests. The total mean costs on each CDCC and non-CDCC participant will be compared using t-tests. The additional costs required for an additional participant in the CDCC Pilot Scheme to achieve target outcomes of disease control after 12 months will be reported as the ICER, calculated as the incremental cost incurred for each participant of the CDCC Pilot Scheme divided by the difference in the proportion of participants meeting targeted disease outcome. Cost-effectiveness of the Scheme would be determined by comparing the ICER to the willing-to-pay threshold of 1 GDP per year.

All statistical analyses will be performed using Stata (version 15.1). A p-value < 0.05 is considered statistically significant.

## Discussion

The CDCC Pilot Scheme is a territory-wide programme to advocate early screening and management of chronic diseases with private-public partnership in the community setting. The implementation of the Scheme involves various stakeholders including the Primary Healthcare Commission, DHC/DHC Express, FDs and allied health professionals. A timely evaluation of the Scheme at this pilot stage is needed to assure the cross-sector collaboration works as expected and to inform strategies for quality enhancement in the future. Guided by the three domains of quality of care (structure, process and outcome), key stakeholders were involved at the initial phase to develop the evaluation criteria and standards, incorporating feedback from service providers based on real-world practice. The inclusion of various stakeholders and providers, and adoption of both subjective (self-reported outcomes) and objective (site visits and health parameters) data collection methods enhance the validity and reliability of the results. Results of the evaluation could shed light on good practices that has already been adopted and inform future guidelines to improve service quality.

Previous evaluation studies on the Risk Assessment and Management Program in Hong Kong, a chronic disease management programme implemented in public primary care, discussed approaches to facilitate the evaluation of multi-disciplinary programme [26]. They include coordinating stakeholder collaboration and communication, continuous feedback and planning meetings, stakeholder endorsement and validation, development of evaluation framework and operation definitions, and ensuring data quality. These recommendations have been incorporated in the current evaluation.

The CDCC Pilot Scheme was officially launched in the November 2023. To date, the evaluation framework has been developed, and have already conducted all site visits to DHC/DHC Express. The recruitment of participants for the participant experience and enablement survey and control group non-participants are in progress. We anticipate the major challenges in the evaluation to be:

1. Stakeholder endorsement and validation.

Standard operation protocols have been established by the Primary Healthcare Commission to specify the standards of operation for DHC/DHC Express and FDs. Since the DHC/DHC Express varied in scale, resources available, and located across districts, slight variations in real-world practice among centers is expected. For instance, staff availability, capacity of the centre, and the number of participants recruited can influence the mode of service delivery. It is essential to understand these potential variations in daily operational practices through site visits and to collectively explore solutions. Core criteria for ensuring standardized care are delivered across centers should be identified, and adherence to such criteria is non-negotiable. Alternatively, criteria that enhances patient care but are outside of the core criteria may effectively inform practices that would improve quality of care once resources become available.

2. Development of evaluation framework and operation definitions.

The indicators and standards of the evaluation framework are derived from standard operation protocols derived by the Primary Healthcare Commission for various service providers. To facilitate comprehension and interpretation of the framework, operation definitions have been developed for the framework, as well as specifying the service providers applicable for each indicator, and the corresponding source of data (site visit, questionnaire or data extraction) in order to ease data collection.

3. Ensuring data quality.

The data of the CDCC Pilot Scheme are captured in multiple IT modules and managed by the Primary Healthcare Commission. Since a range of personnel from different service providers can input and access participant data through the IT modules, monitoring data quality is of utmost importance for a valid and reliable evaluation. To achieve this, we have worked closely with the Commission to align our understanding of the data fields and their definitions. Several issues observed to date have included (i) incomplete data entry such as laboratory records, missing attendances and treatment plans, (ii) missing diagnoses of participants who are lost to follow-up after the initial screening phase, and (iii) missing consultation records by FDs who encounter difficulties using the IT system. To overcome these challenges, through discussion with the Commission and service operators, (i) participants' diagnosis will be defined by the latest treatment plan, the latest laboratory record will be used for evaluating effectiveness. Staff at DHC/DHC Express will also (ii) follow up with the FDs on the missing diagnosis and (iii) provide guidance on the system interface to the FDs who are in need.

Maintaining close collaboration and communication with stakeholders is the key to implementing this evaluation study. We shall gather their feedback through regular meetings and address the concerns from different parties, including the expectation of the health policymakers, the progress of deliverables achievement by service operators, and the practical difficulties encountered by frontline staff. The goal of this evaluation study is to consolidate the issues that arose in practice and advise on feasible solutions upon discussion with relevant stakeholders, with the aim of enhancing the capacity of providers to offer quality care and raise participant satisfaction. Results from this evaluation would provide practical and specific recommendations to inform optimal delivery of the CDCC Pilot Scheme, while the processes used to develop this evaluation can be easily adapted for similar cross-sector screening and management programme in the community to improve the delivery of primary healthcare services.

## Electronic supplementary material

Below is the link to the electronic supplementary material.

Supplementary Material 1: **Appendix A:** Evaluation framework for the Chronic Disease Co-Care (CDCC) Pilot Scheme.

Supplementary Material 2: **Appendix B:** Participant satisfaction and enablement survey for the Chronic Disease Co-Care Pilot Scheme at 3 months post-enrolment.

Supplementary Material 3: **Appendix C:** Costing questionnaire for District Health Centre (Expresses) over 1 year for the Chronic Disease Co-Care Pilot Scheme.

Supplementary Material 4: **Appendix D**: Participant satisfaction, enablement, and healthcare utilization survey for the Chronic Disease Co-Care Pilot Scheme at 12 months post-enrolment.

## Data Availability

The data that support the findings of this study are available from the Primary Healthcare Commission, Health Bureau, the Government of Hong Kong and the Hong Kong Hospital Authority, but restrictions apply to the availability of these data, which were used under license for the current study, and so are not publicly available.

## Abbreviations

BP: Blood pressure
CDCC: Chronic Disease Co-Care
DHC: District Health Centre
DM: Diabetes mellitus
FD: Family doctor
GDP: Gross domestic product
HbA1c: Glycated hemoglobin
HT: Hypertension
ICER: Incremental cost-effectiveness ratio
PEI: Patient Enablement Instrument
PHC: Primary healthcare
Pre-DM: Pre-diabetes
