# Supplementary material for: Evaluation of the effectiveness and cost-effectiveness of the chronic disease co-care (CDCC) Pilot Scheme: a study protocol
Source: BMC Prim Care. 2025 Mar 19;26:73. doi: 10.1186/s12875-025-02765-6 (PMC11921508; doi:10.1186/s12875-025-02765-6)
Supplement: Supplementary file 1 — Supplementary Material 1: Appendix A: Evaluation framework for the Chronic Disease Co-Care (CDCC) Pilot Scheme. [file 12875_2025_2765_MOESM1_ESM.pdf]

Appendix A: Evaluation framework for the Chronic Disease Co-Care (CDCC) Pilot Scheme

Human Resources		Target Std	Service provider	Source
1.	There **must** be designated coordinator(s) to oversee the CDCC-Pilot Scheme in the Primary Healthcare Office (PHO).	100%	PHO	SOC Qn
2.	There **must** be designated coordinator(s) to oversee the CDCC-Pilot Scheme at each District Health Centre (DHC) / District Health Centre Express (DHCE).	100%	DHC(E)	SOC Qn
3.	There **must** be a multidisciplinary team of health care personnel, including Scheme Doctor(s), nurse(s), physiotherapist(s), dietitian(s), optometrist(s), podiatrist(s), and supporting staff to implement the scheme.<br>*(Health care personnel can either be in-house staff or Network Medical Practitioners (NMP) Network Service Providers (NSP)<br>Please specify the number of programme staff and their time dedicated to the programme.)*	100%	DHC(E)	Site visit & SOC Qn
4.	Nurse(s) and allied health professional(s), including physiotherapist(s), dietitian(s), optometrist(s) and podiatrist(s), **must** be accessible to scheme participants when indicated.<br>*("accessible to" mean able to be visited by)*	100%	Nurse, AH (NSP or DHC(E))	Site visit & SOC Qn
5.	Specialist(s) at the Hospital Authority **must** be accessible to participants for one-off bi-directional specialist consultation service(s) when indicated.<br>*("accessible to" means able to be visited by)*	100%	HA / DHC(E)	Site visit & SOC Qn
6.	DHC/DHCE staff, scheme doctors, nurses and allied health professionals **must** be familiar with the CDCC-Pilot Scheme's objectives, service manual, clinical workflow including referral pathway, IT system and payment system.<br>*("familiar" means being able to provide an overview of the scheme's objectives and logistics<br>Please attach a summary.)*	100%	DHC(E), FD, Nurse, AH	Site visit & SOC Qn
7.	Scheme doctors and nurses **must** know the Hong Kong Reference Frameworks on life course preventive care, diabetes care and hypertension care for adults in primary care setting.<br>*("know" means having read and understand the relevant Hong Kong Reference Frameworks)*	100%	FD, Nurse	Site visit & SOC Qn
8.	DHC/DHCE staff, scheme doctors, nurses and allied health professionals **must** have undergone relevant training to the scheme.	100%	DHC(E), FD, Nurse, AH	Site visit & SOC Qn
Office Infrastructure		Target Std	Service provider	Source
9.	DHC/DHCE staff, scheme doctors, nurses and allied health professionals **must** have authorised access to the eHealth system.<br>*("authorized access" means being able to use the system)*	100%	DHC(E), FD, Nurse, AH	Site visit & SOC Qn
10.	Scheme doctors, nurses and allied health professionals **must** have authorised access to the CDCC-IT system for participant data entry, review, sharing and retrieval.	100%	FD, Nurse, AH	Site visit & SOC Qn

11.	The CDCC-IT system **must** be used for participant enrolment, and documentation of participant clinical data and management plan rendered under the CDCC-pilot scheme.	100%	DHC(E), FD, Nurse, AH	Site visit & SOC Qn
12.	Scheme doctors **must** use the CDCC-IT system for making referral(s) and/or consultation(s) to specialists in the Hospital Authority and allied health professionals in the scheme.	100%	FD	Site visit & SOC Qn
13.	A booking system **must** be available for DHC/DHCE staff to arrange appointment(s) between participants and scheme doctors, nurses and allied health professionals.	100%	DHC(E), FD, Nurse, AH	Site visit
14.	A reminder system **must** be available for nurse(s) to remind participants for investigations and appointments with scheme doctor and allied health professionals.	100%	Nurse	Site visit
15.	There **must** be appropriate physical space provided for the Scheme.<br>*("appropriate" means having dedicated space to allow for smooth operation of the Scheme; this could include space for participant registration, performing the HRFA and nurse clinic sessions)*	100%	DHC(E), FD, Nurse, AH	Site visit
16.	Blood Pressure measurement device(s) **must** be available in DHC/DHCE and clinic(s) of the Scheme doctors.	100%	DHC(E), FD	Site visit & SOC Qn
17.	Instrument for testing sensation of the feet (e.g. tuning fork or monofilaments) **must** be available in the nurse clinic.<br>*(note: having either the tuning fork or monofilament is considered adequate.)*	100%	Nurse	SOC Qn
18.	Educational material(s), computer(s) and printer(s) **must** be available at all CDCC-pilot scheme service points.	100%	DHC(E), FD, Nurse, AH	Site visit
19.	Fundi camera for retinal photo **must** be accessible to participants when indicated.	100%	AH (optometrist)	SOC Qn
20.	Scheme doctors **must** have access to designated laboratory services for the CDCC Pilot Scheme.<br>*("have access to" means being able to order laboratory service)*	100%	FD	SOC Qn
21.	Laboratory test results **must** be available for viewing online by scheme doctors / nurses within 7 working days from date of testing.	100%	FD, Nurse	Site visit & SOC Qn
22.	A reminder system **must** be available to notify the scheme doctors after the laboratory test results have been uploaded online.	100%	FD	Site visit & SOC Qn
23.	An emergency recall system **must** be available for laboratories to inform scheme doctors / nurses of critical results.	100%	FD, Nurse	Site visit & SOC Qn
24.	Scheme doctors **must** have access to drugs on the list of specified drugs for the CDCC-Pilot Scheme.<br>*("have access to" means being able to prescribe)*	100%	FD	Site visit & SOC Qn

25.	A scheme enquiry system must be available for scheme doctors, nurses and allied health professionals.	100%	DHC(E), FD, Nurse, AH, ST	Site visit & SOC Qn
26.	A scheme enquiry system must be available for scheme participants.	100%	DHC(E)	Site visit & SOC Qn
Programme Management & Organizational Structure		Target Std	Service provider	Source
27.	Participant's enrolment and pairing record with the scheme doctor of choice **must** be properly documented and accessible in the eHealth/CDCC-IT system for authorised members of the scheme.	100%	DHC(E), FD, Nurse, AH	SOC Qn
28.	Co-payment amount for each scheme doctor, allied health services and laboratory testing **must** be accessible to participants.<br>*("accessible to" means being to)*	100%	DHC(E), FD, Nurse, AH	SOC Qn
29.	There **must** be regular meetings among staff involved in the implementation of the scheme at each DHC/DHCE to monitor the performance of the programme.	100%	DHC(E)	SOC Qn
30.	There **must** be regular meetings among CDCC operation teams in DHC/DHCE and PHO to monitor the performance of the programme.	100%	DHC(E), PHO	SOC Qn

Process evaluation framework

Patient and service provider eligibility		Target Std	Source
1.	Participants enrolled into the CDCC-Pilot Scheme **must** be HK residents aged ≥45 years and without known diabetes mellitus (DM) or hypertension (HT).	100%	CDCC IT Module
2.	Participants **must** be a member of DHC/DHCE.	100%	CDCC IT Module
3.	Participants **must** agree to sharing their data in the electronic health record sharing system (eHealth).	100%	CDCC IT Module
4.	Participants **must** have consented to CDCC-Pilot Scheme Terms & Conditions.	100%	SOC Qn CDCC IT Module
5.	Participants **must** be paired with a Family Doctor.	100%	CDCC IT Module
6.	Scheme doctors **must** be registered in the Primary Care Doctor (PCD) directory.	100%	Site visit & SOC Qn
7.	Scheme doctors **must** have consented to CDCC-Pilot Scheme Terms & Conditions.	100%	Site visit & SOC Qn
Service Delivery & Process of Care		Target Std	Source
8.	Participants **must** have at least one Blood Pressure (BP) measurement at the scheme doctor's office.	100%	CDCC IT Module
9.	Participants **must** have at least one Haemoglobin-A1c (HbA1c) or fasting plasma glucose (FPG) measurement.	100%	CDCC IT Module
10.	Participants **should** have at least one subsidised medical consultation within 12 months from the screening date.<br><br>*("screening date": the date of 1st screening consultation)*	70%	CDCC IT Module
11.	Participants who were started on medication treatment **should** have at least two subsidised medical consultation within 12 months from the screening date.<br><br>*("screening date": the date of 1st screening consultation)*	70%	CDCC IT Module
12.	Participants **should** have one nurse clinic session within 3 months from the start of treatment phase.<br><br>*("start of treatment phase": the date of 1st medical consultation)*	70%	CDCC IT Module
13.	Participants **should** have at least one Health Risk Factor Assessment (HRFA) within 12 months the screening date.<br><br>*("screening date": the date of 1st screening consultation)*	70%	DHC IT Module, CDCC IT Module
14.	Participants **should** have at least one repeat HbA1c or FPG measurement within 12 months from the screening date.<br><br>*("screening date": the date of 1st screening consultation)*	70%	CDCC IT Module

15.	Participants **should** have at least one repeat full lipid profile measurement 12 months from the screening date.<br><br>*("screening date": the date of 1st screening consultation)*	70%	CDCC IT Module
16.	Participants **could** have one Intensive Diabetes Prevention Programme (IDPP) session within 6 months from the start of treatment phase.<br><br>*("start of treatment phase": the date of 1st medical consultation)*	50%	CDCC IT Module
17.	Participants **should** have at least one full lipid profile measurement within 3 months from the screening date.<br><br>*("screening date": the date of 1st screening consultation)*	90%	CDCC IT Module
18.	Participants **should** have at least one renal function test (RFT) with glomerular filtration rate (eGFR) results within 3 months from the screening date.<br><br>*("screening date": the date of 1st screening consultation)*	90%	CDCC IT Module
19.	Participants with HT **should** have at least one urine analysis measurement within 3 months from the screening date.<br><br>*("screening date": the date of 1st screening consultation)*	90%	CDCC IT Module
20.	Participants with DM **should** have at least one urine albumin-creatinine ratio (ACR) measurement within 3 months from the screening date.<br><br>*("screening date": the date of 1st screening consultation)*	90%	CDCC IT Module
21.	Participants **should** have at least one foot assessment within 3 months from the start of treatment phase.<br><br>*"start of treatment phase": the date of 1st medical consultation)*	90%	CDCC IT Module
22.	Participants **should** have at least one optometry assessment within 3 months from the start of treatment phase.<br><br>*("start of treatment phase": the date of 1st medical consultation)*	90%	CDCC IT Module
23.	Participants **should** have at least two subsidised medical consultations within 12 months from the screening date.<br><br>*("screening date": the date of 1st screening consultation)*	90%	CDCC IT Module
24.	Participants started on medication treatment **must** have at least four subsidised medical consultations within 12 months from the screening date.<br><br>*("screening date": the date of 1st screening consultation)*	90%	CDCC IT Module
25.	Participants **should** have at least one nurse clinic session within 3 months from the start of treatment phase.<br><br>*("start of treatment phase": the date of 1st medical consultation)*	70%	CDCC IT Module
26.	Participants **should** have at least one HRFA within 12 months from the screening date.<br><br>*("screening date": the date of 1st screening consultation)*	70%	DHC IT Module, CDCC IT Module

27.	Participants with HT **should** have at least one repeat HbA1c or FPG measurement within 12 months from the screening date.<br><br>*("screening date": the date of 1st screening consultation)*	70%	CDCC IT Module
28.	Participants with DM **should** have at least one repeat HbA1c measurement within 12 months from screening date.<br><br>*("screening date": the date of 1st screening consultation)*	70%	CDCC IT Module
29.	Participants **should** have at least one repeat full lipid profile measurement within 12 months from screening date.<br><br>*("screening date": the date of 1st screening consultation)*	70%	CDCC IT Module
30.	Participants **should** have at least one repeat RFT with eGFR result within 12 months from the screening date.<br><br>*("screening date": the date of 1st screening consultation)*	70%	CDCC IT Module
31.	Participants with HT **should** have at least one repeat urine analysis within 12 months from the screening date.<br><br>*("screening date": the date of 1st screening consultation)*	70%	CDCC IT Module
32.	Participants with DM **should** have at least one repeat urine ACR measurement within 12 months from the screening date.<br><br>*("screening date": the date of 1st screening consultation)*	70%	CDCC IT Module
33.	Participants **should** have at least one repeat foot assessment within 12 months from the screening date.<br><br>*("screening date": the date of 1st screening consultation)*	70%	CDCC IT Module
34.	Participants with DM **should** have at least one repeat optometry assessment within 12 months from the screening date.<br><br>*("screening date": the date of 1st screening consultation)*	70%	CDCC IT Module
35.	Participants **could** have at least one Patient Empowerment Programme (PEP) session within 6 months from the start of treatment phase.<br><br>*("start of treatment phase": the date of 1st medical consultation)*	50%	CDCC IT Module
Service Delivery & Process of Care		Target Std	Source
**Life course preventive care (All participants)**			
(i)	Vaccination		
1.	Participants **could** be given advice on seasonal influenza vaccine	50%	CDCC IT Module
2.	Participants aged 65 years and above **could** be given advice on pneumococcal vaccine.	50%	CDCC IT Module
(ii)	Cancer Screening		
3.	Participants aged 50 to 75 **could** be given advice on colorectal cancer screening.	50%	CDCC IT Module

4.	Female participants aged 45 and above **<u>could</u>** be given advice on cervical cancer screening.	50%	CDCC IT Module
5.	Female participants aged 45 to 69 **<u>could</u>** be given advice on Breast cancer screening.	50%	CDCC IT Module
(iii)	Lifestyle modification activities		
6.	Current smokers **<u>could</u>** receive at least one intervention under the topic "Smoking Cessation".	30%	CDCC IT Module, DHC IT Module
7.	Drinkers **<u>could</u>** receive at least one intervention under the topic "Alcohol Abstinence".	30%	CDCC IT Module, DHC IT Module
8.	Participants with BMI ≥ 25 kg/m^2 **<u>could</u>** receive at least one intervention under the topic "Weight Management".	30%	CDCC IT Module, DHC IT Module

Outcome evaluation framework

Clinical Outcomes		Target Std	Source
1.	Participants with DM **should** have HbA1c < 7% one year after joining the scheme.<br>(Incentive Payment to Dr (1): ≥ 70% Scheme Participants achieved the HbA1c < 7%)	70%	CDCC IT Module
2.	Participants with DM **should** have improvement in HbA1c 12 months after joining the scheme.	70%	CDCC IT Module
3.	Participants with DM **should** have BP < 130/80 mmHg 12 months after joining the scheme.<br>(Incentive Payment to Dr (2) : ≥ 70% Scheme Participants with DM achieved the BP < 130/80 mmHg)	70%	CDCC IT Module
4.	Participants with HT without DM **should** have BP < 140/90 mmHg 12 months after joining the scheme.<br>(Incentive Payment to Dr (3) ≥ 70% Scheme Participants with HT without DM achieved the BP < 140/90 mmHg)	70%	CDCC IT Module
5.	Participants with HT **should** have improvement in BP 12 months after joining the scheme.	70%	CDCC IT Module
6.	Participants with DM **should** have Low Density Lipoprotein-Cholesterol (LDC-C) <2.6 mmol/L 12 months after joining the scheme.	70%	CDCC IT Module
7.	Participants with DM **could** have improvement in LDC-C 12 months after joining the scheme.	50%	CDCC IT Module
8.	Participants with Body Mass Index (BMI) ≥ 25 kg/m^2 **could** have improvement in BMI 12 months after joining the scheme.	50%	CDCC IT Module
9.	Number of smokers **could** decrease 12 months after the scheme	Decrease	CDCC IT Module
10.	Number of alcohol drinkers **could** decrease 12 months after the scheme.	Decrease	CDCC IT Module
11.	Number of participants with BMI ≥ 25 kg/m^2 **could** decrease 12 months after the scheme.	Decrease	CDCC IT Module

Patient-reported outcomes			Source
12.	Patients **<u>should</u>** be more enabled after joining the scheme.	70%	PRO Qn
13.	Patients **<u>could</u>** have home BP monitoring and report in eHealth App at least once per month	50%	eHRSS
