# Supplementary material for: Evaluation of the effectiveness and cost-effectiveness of the chronic disease co-care (CDCC) Pilot Scheme: a study protocol
Source: BMC Prim Care. 2025 Mar 19;26:73. doi: 10.1186/s12875-025-02765-6 (PMC11921508; doi:10.1186/s12875-025-02765-6)
Supplement: Supplementary file 2 — Supplementary Material 2: Appendix B: Participant satisfaction and enablement survey for the Chronic Disease Co-Care Pilot Scheme at 3 months post-enrolment. [file 12875_2025_2765_MOESM2_ESM.pdf]

Appendix B: Participant satisfaction and enablement survey for the Chronic Disease Co-Care Pilot Scheme at 3 months post-enrolment

Participant ID: ____________________________ **Date:** ____________________________ (DD/MM/YYYY)

Please check the appropriate box.

**1.**	**Enrolment process at DHC/DHCE**	**Definitely**	**Mostly**	**Somewhat**	**Not at all**
1.1	The opening hours for the DHC/DHCE or clinic are convenient	☐	☐	☐	☐
1.2	The location of the DHC/DHCE or clinic is convenient and easily accessible	☐	☐	☐	☐
1.3	There were sufficient choices for family doctors on the Primary Care Directory	☐	☐	☐	☐
1.4	The DHC staff/nurse provided me with adequate information regarding the steps and components of the CDCC Pilot Scheme	☐	☐	☐	☐
1.5	The DHC staff/nurse provided me with clear information about payments, subsidy amounts and incentive mechanisms of the CDCC Pilot Scheme	☐	☐	☐	☐

**2.**	**Care and treatment at and/or at the first family doctor consultation**	**Definitely**	**Mostly**	**Somewhat**	**Not at all**
2.1	I feel that I had to wait a long time to get an appointment at my desired family doctor	☐	☐	☐	☐
2.2	I feel that I had to wait a long time between completing the administrative procedures and seeing the doctor (i.e. waiting in queue in reception)	☐	☐	☐	☐
2.3	The doctor provided clear and understandable answers when I had questions	☐	☐	☐	☐
2.4	The doctor explained my diagnosis, test results and treatment in a way I could understand	☐	☐	☐	☐
2.5	The doctor involved me in decisions about my care and treatment (e.g. the doctor respected my values and preferences when they recommended treatments to me)	☐	☐	☐	☐
2.6	The doctor listened carefully to me during my visit	☐	☐	☐	☐
2.7	The doctor helped me set specific goals to improve my lifestyle habits	☐	☐	☐	☐
2.8	The doctor considers all factors that affect my health in caring for me (e.g. knowledge of my family and my community)	☐	☐	☐	☐
2.9	I feel that the doctor spent enough time with me during my visit	☐	☐	☐	☐

2.10	Were you referred to allied health professionals and/or specific programs by your FD?<br>☐ Yes<br>☐ No (proceed to 2.12)				
2.11	I feel that the referral was necessary and will be beneficial for my condition	☐	☐	☐	☐
2.12	The doctor explained how multi-disciplinary care could help my treatment	☐	☐	☐	☐
2.13	Were you prescribed any medications by your FD?<br>☐ Yes<br>☐ No (proceed to part 3)				
2.14	The doctor/nurse told me in a clear and understandable way on how to take my medications	☐	☐	☐	☐
2.15	The doctor/nurse explained to me the effects of the medication in a way I could understand	☐	☐	☐	☐
2.16	I will be willing to continue visiting the same family doctor	☐	☐	☐	☐
2.17	I have confidence and trust in the family doctor treating me	☐	☐	☐	☐

2.18 Using any number from 0 to 10, where 0 is the worst visit possible and 10 is the best visit possible, what number would to use to rate your first visit with your FD?

0	1	2	3	4	5	6	7	8	9	10
☐	☐	☐	☐	☐	☐	☐	☐	☐	☐	☐
Worst visit possible										Best visit possible

**3.**	**Follow-up laboratory investigations**	**Definitely**	**Mostly**	**Somewhat**	**Not at all**
3.1	The opening hours for the laboratories are convenient	☐	☐	☐	☐
3.2	The location of the laboratories is convenient and easily accessible	☐	☐	☐	☐
3.3	I feel that I had to wait a long time to get an appointment for the laboratory investigations	☐	☐	☐	☐
3.4	I feel that I had to wait a long time to receive the blood tests at the laboratories (i.e. waiting in queue in reception)	☐	☐	☐	☐
3.5	The laboratory staff treated me in a professional manner	☐	☐	☐	☐
3.6	The laboratory staff provided clear and understandable answers to me when I had questions	☐	☐	☐	☐

4.	Patient Enablement: After the first doctor consultation, I feel I am:	Greatly improved	Slightly improved	The same or less	N/A
4.1	able to face my life	☐	☐	☐	☐
4.2	able to understand my illness	☐	☐	☐	☐
4.3	able to live with my illness	☐	☐	☐	☐
4.4	able to maintain good health	☐	☐	☐	☐
4.5	confident about my health	☐	☐	☐	☐
4.6	able to self-help	☐	☐	☐	☐

5.	Payment	Definitely	Mostly	Somewhat	Not at all
5.1	I am happy with the subsidy coverage provided in this Scheme.	☐	☐	☐	☐
5.2	The amount charged for the screening process is reasonable	☐	☐	☐	☐
5.3	The amount charged for the FD consultation is reasonable	☐	☐	☐	☐
5.4	The amount charged for the medications is reasonable *(only for patients who were prescribed any medications)*	☐	☐	☐	☐
5.5	The doctor-patient partnership incentive mechanism provided extra motivation for me to better care for my health	☐	☐	☐	☐

6.	Overall impression	Definitely	Mostly	Somewhat	Not at all
6.1	The CDCC Pilot Scheme makes it easy for me to get medical care	☐	☐	☐	☐
6.2	The CDCC Pilot Scheme is able to provide most of the medical care I need	☐	☐	☐	☐
6.3	I felt more motivated to better care for my health after joining the CDCC Pilot Scheme	☐	☐	☐	☐
6.4	Overall, joining the CDCC Pilot Scheme helps me meet my health-related goals	☐	☐	☐	☐
6.5	Overall, joining the CDCC Pilot Scheme helps me stay healthy	☐	☐	☐	☐
