# Supplementary material for: Evaluation of the effectiveness and cost-effectiveness of the chronic disease co-care (CDCC) Pilot Scheme: a study protocol
Source: BMC Prim Care. 2025 Mar 19;26:73. doi: 10.1186/s12875-025-02765-6 (PMC11921508; doi:10.1186/s12875-025-02765-6)
Supplement: Supplementary file 3 — Supplementary Material 3: Appendix C: Costing questionnaire for District Health Centre (Expresses) over 1 year for the Chronic Disease Co-Care Pilot Scheme. [file 12875_2025_2765_MOESM3_ESM.pdf]

Appendix C: Costing questionnaire for District Health Centre (Expresses) over 1 year for the Chronic Disease Co-Care Pilot Scheme

This questionnaire aims to collect information on the type and amount of resources used on CDCC in your DHC/DHCE. For each type of expense, please provide the units of items used and/or their estimated monetary value where applicable.

**To be completed by Executive directors/Chief Care Co-ordinators**

Centre: ______________________

Date: ______________________

We might need to contact you again for further questions. If this would be acceptable, please provide your contact details below:

E-mail: _________________

Telephone: ______________

If there are any questions on completing the questionnaire, please do not hesitate to contact Dr. Eric Wan at 2831-5057 (yfwan@hku.hk).

Please indicate the **number of participants enrolled (participant-count, not attendance)** into the CDCC in your DHC/DHCE from **January 1st 2024 to December 31st 2024**

ONE year from Jan 2024 to Dec 2024: _________ participants

**Part A: Set-up costs**	
1.1	Did your DHC/DHCE provide any training for the staff to deliver CDCC?<br><br>☐ Yes ☐ No (please go to 2.1)
1.2	**If yes**, please indicate the **total cost of resources** (including trainers and materials used) needed for providing the training in your DHC/DHCE, from **Jan 2024 to Dec 2024**?<br><br>HK$__________________
1.3	Please indicate the **rank and number who attended,** and the **training time** for both trainers and trainees in total from **Jan 2024 to Dec 2024**:<br><br>For example: There are **2 Registered nurses** training **3 Patient Care Assistants in a 4-hour session.** The information will be filled as below.<br>Rank of staff — Total training time in one year<br>Registered Nurse(s) — *2×4=8* Hours<br>Patient Care Assistant(s); — *3×4=12* Hours<br><br>Rank of staff — Total training time in one year<br>______________ — ______________ Hours<br>______________ — ______________ Hours<br>______________ — ______________ Hours<br>______________ — ______________ Hours<br>______________ — ______________ Hours

2.1	Did your DHC/DHCE use any resources (*e.g. educational tool/material*) **specifically** for **setting up the CDCC**?<br><br>☐ Yes ☐ No (please go to 3.1)
2.2	If yes, please indicate the **type of** resources and **total cost** of each for **setting up CDCC** in your

	DHC/DHCE, between **<u>Jan 2024 and Dec 2024</u>**:<br><br><u>Type of resources</u> <u>Total cost in one year</u><br><br>________________________________ HK$_______________<br>________________________________ HK$_______________<br>________________________________ HK$_______________<br>________________________________ HK$_______________<br>________________________________ HK$_______________
3.1	Were there any **other resources** (e.g. infrastructure and renovation work) used for **setting up the CDCC** in your DHC/DHCE?<br><br>☐ Yes ☐ No (please go to Part B)
3.2	If yes, please indicate the **type of resources** and **total cost** for **setting up CDCC** in your DHC/DHCE, from **<u>Jan 2024 to Dec 2024</u>**:<br><br><u>Type of expense</u> <u>Total cost in one year</u><br><br>________________ HK$________________<br>________________ HK$________________<br>________________ HK$________________

Part B: On-going recurrent costs
**Staff and resources used by CDCC in your DHC/DHC Express from Jan 2024 to Dec 2024**

Operation Definitions:

a) Recruitment and intake assessment

- Screening for eligibility (including assistance provided to participants on DHC/DHCE member registration, seeking informed consent on data sharing, and explaining terms and conditions of CDCC Scheme), conducting Health risk factors assessment (HRFA) for eligible CDCC participants (data collection on demographics, social background, family history, past medical history, medication, lifestyle behaviour, physical examination and psychological health status), and facilitating family doctor matching

b) Lifestyle intervention

- Organising lifestyle modification activities as well as providing patient empowerment programmes

Recruitment and intake assessment [a]

1.1 Please indicate the **average number of individuals being screened for eligibility**, and **assessed** per month [a] in your DHC/DHCE, from **Jan 2024 to Dec 2024**:

Eligibility screening:_________ individuals per month

Intake assessment:_________ individuals per month

[a] Please refer to the "Operation Definition" at the beginning of the questionnaire.

1.2 What is the **average duration (in minute)** per **eligibility screening** and **intake assessment** for each CDCC participant in your DHC/DHCE, from **Jan 2024 to Dec 2024**:

Eligibility screening: ___________ (average) minutes per CDCC participant

Intake assessment: ___________ (average) minutes per CDCC participant

1.3 Please indicate the **number, rank, average time (in minute) spent per patient, and hourly rate of staff** needed to operate the **recruitment** and **intake assessment** [a] *(including preparation work*):

	Rank	No. of staff	Average time spent per patient	Hourly rate (HKD$)
☐	_________________	_______	______________ minutes	____________
☐	_________________	_______	______________ minutes	____________
☐	_________________	_______	______________ minutes	____________

	[a] Please refer to the "Operation Definition" at the beginning of the questionnaire.
1.4	Please indicate the **type of resources** (*e.g. printing and consumable items*) and the **total cost** of each type to operate the **recruitment and intake assessment** [a] (*include preparation work*) from **Jan 2024 to Dec 2024**:<br><br>Type of expense — Total cost in one year<br>__________ HK $_____<br>__________ HK $_____<br>__________ HK $_____<br>__________ HK $_____<br>__________ HK $_____<br>[a] Please refer to the "Operation Definition" at the beginning of the questionnaire.

**Lifestyle intervention** [b]	
2.1	Please indicate the **number, rank, average time (in minute) spent per participant, and hourly rate of staff** needed to operate the **lifestyle intervention programme** [b] *(including preparation work*):<br><br>Rank | No. of staff | Average time spent per participant | Hourly rate (HKD$)<br>☐ _____ | _____ | _____ minutes | _____<br>☐ _____ | _____ | _____ minutes | _____<br>☐ _____ | _____ | _____ minutes | _____<br>☐ _____ | _____ | _____ | _____<br><br>[b] Please refer to the "Operation Definition" at the beginning of the questionnaire.
2.2	Please indicate the **type of resources** (*e.g. printing and consumable items*) and the **total cost** of each type to operate the **lifestyle intervention** [a] (*include preparation work*) from **Jan 2024 to Dec 2024**:<br><br>Type of expense — Total cost in one year<br>__________ HK $_____<br>__________ HK $_____<br>__________ HK $_____<br>__________ HK $_____<br>__________ HK $_____<br>[b] Please refer to the "Operation Definition" at the beginning of the questionnaire.

Part C: Administrative or Supportive tasks	
1.	**Apart from the staff involved in questions 1.3 and 2.1**, please indicate the **number, rank, average time (min) spent per participant, and hourly rate of staff** who provided **administrative or supportive tasks** of **CDCC per month on average** from **Jan 2024 to Dec 2024**:<br><br>Rank / Number / Average time spent per participant per month / Hourly rate (HKD$)<br>☐ ________ ________ ________ minutes ________<br>☐ ________ ________ ________ minutes ________<br>☐ ________ ________ ________ minutes ________<br>☐ ________ ________ ________ minutes ________
**Other expenses**	
1.1	Are there any other **on-going resources** used for CDCC in your DHC/DHCE? *(exclude the expenses on utilities such as electricity, water, etc)*<br><br>☐ Yes ☐ No (End)
1.2	If yes, please indicate the **type of resources** and **total cost of each** for CDCC in your centre from **Jan 2024 to Dec 2024**:<br><br>Type of expense / Total cost in one year<br>________ HK $________<br>________ HK $________<br>________ HK $________<br>________ HK $________<br>________ HK $________

-THE END-

Thank you very much!
