# Supplementary material for: Evaluation of the effectiveness and cost-effectiveness of the chronic disease co-care (CDCC) Pilot Scheme: a study protocol
Source: BMC Prim Care. 2025 Mar 19;26:73. doi: 10.1186/s12875-025-02765-6 (PMC11921508; doi:10.1186/s12875-025-02765-6)
Supplement: Supplementary file 4 — Supplementary Material 4: Appendix D: Participant satisfaction, enablement, and healthcare utilization survey for the Chronic Disease Co-Care Pilot Scheme at 12 months post-enrolment. [file 12875_2025_2765_MOESM4_ESM.pdf]

Appendix D: Participant satisfaction, enablement, and healthcare utilization survey for the Chronic Disease Co-Care Pilot Scheme at 12 months post-enrolment

Participant ID: ____________________________ **Date:** ____________________________
(DD/MM/YYYY)

Think about your experience while receiving medical care under the CDCC Pilot Scheme over the past 12 months:

**1.**	**Family doctor consultation**	**Definitely**	**Mostly**	**Somewhat**	**Not at all**
1.1	Of all doctor consultations at primary care providers over the past 12 months, of what proportion did you see the FD paired up in this Scheme?<br>☐ 0 - 25%<br>☐ 26 – 50%<br>☐ 51 – 75%<br>☐ 76 – 100%				
1.2	I feel that I had to wait a long time to get an appointment with my FD every time	☐	☐	☐	☐
1.3	On average, how long did you have to wait for your appointment with your FD?	_________ days / _________ weeks			
1.4	I was able to get an appointment with my FD whenever I need it	☐	☐	☐	☐
1.5	I was given enough time to communicate with my doctor at each appointment	☐	☐	☐	☐
1.6	The doctor provided clear and understandable answers when I had questions	☐	☐	☐	☐
1.7	The FD consultations were essential and useful for managing my condition	☐	☐	☐	☐
1.8	I would benefit more from the programme if the FD consultations were more frequent	☐	☐	☐	☐
1.9	I had confidence and trust in the FD treating me	☐	☐	☐	☐
1.10	The doctor involved me in decisions about my care and treatment	☐	☐	☐	☐
1.11	It is important for me to meet the same FD at every visit	☐	☐	☐	☐
1.12	The amount charged for the FD consultation is reasonable	☐	☐	☐	☐
1.13	My FD helped me identify resources in my community (e.g. classes, community programmes)	☐	☐	☐	☐

2.	**Nurse clinic follow-up**	**Definitely**	**Mostly**	**Somewhat**	**Not at all**
2.1	I feel that I had to wait a long time to get an appointment with my nurse every time	☐	☐	☐	☐
2.2	On average, how long did you have to wait for your appointment for the nurse follow-up?	_________ days / _________ weeks			
2.3	I was able to get an appointment for the nurse follow-up whenever I need it.	☐	☐	☐	☐
2.4	I was given sufficient time at each appointment at the nurse clinic	☐	☐	☐	☐
2.5	The nurse provided clear and understandable answers when I had questions	☐	☐	☐	☐
2.6	The nurse clinic(s) were essential and useful for managing my condition	☐	☐	☐	☐
2.7	I would benefit more from the programme if the nurse clinic sessions were offered more frequently	☐	☐	☐	☐
2.8	I had confidence and trust in the nurses treating me	☐	☐	☐	☐
2.9	The nurses involved me in decisions about my care and treatment	☐	☐	☐	☐
2.10	The nurse health clinic(s) provided me with new skills in caring for myself	☐	☐	☐	☐
2.11	The location of the nurse clinic(s) I was referred to was convenient and easily accessible	☐	☐	☐	☐

3.	**Allied health professionals**	**Definitely**	**Mostly**	**Somewhat**	**Not at all**
3.1	I feel that I had to wait a long time to get an appointment with my allied health professionals every time	☐	☐	☐	☐
3.2	On average, how long did you have to wait for your appointment with your allied health professionals?	_________ days / _________ weeks			
3.3	I was able to get an appointment with my allied health professional whenever I need it.	☐	☐	☐	☐
3.4	I was given sufficient time at each appointment with the allied health professionals	☐	☐	☐	☐
3.5	The allied health professionals staff provided clear and understandable answers when I had questions	☐	☐	☐	☐
3.6	The allied health professionals were essential and useful for managing my condition	☐	☐	☐	☐
3.7	I would benefit more from the programme if allied health sessions were offered more frequently	☐	☐	☐	☐
3.8	I had confidence and trust in the allied health professionals treating me	☐	☐	☐	☐

3.9	The allied health professionals involved me in decisions about my care and treatment	☐	☐	☐	☐
3.10	The location of allied health professionals I was referred to were convenient and easily accessible	☐	☐	☐	☐
3.11	The amount charged for the allied health professional is reasonable	☐	☐	☐	☐

**4.**	**DHC/DHC Express activities and empowerment programme**	**Definitely**	**Mostly**	**Somewhat**	**Not at all**
4.1	What DHC / DHCE activities and empowerment programme(s) have you attended?<br>☐ Intensive diabetes management programme (IDDP)<br>☐ PEP<br>☐ Others, please specify: ________________<br>☐ I have not attended any in the past 12 months (proceed to part 5)				
4.2	The programme staff provided clear and understandable answers when I had questions	☐	☐	☐	☐
4.3	The programme was essential and useful for managing my condition	☐	☐	☐	☐
4.4	The programme helped me understand what to do to manage my condition	☐	☐	☐	☐
4.5	The programme provided information about behaviour change and problem-solving strategies about my condition	☐	☐	☐	☐
4.6	The programme provided practical strategies to change my lifestyle behaviours to improve my condition	☐	☐	☐	☐
4.7	The programme supported and facilitated self-management of my condition	☐	☐	☐	☐
4.8	I had confidence and trust in the programme staff delivering the programme	☐	☐	☐	☐
4.9	The programme was offered at a location that was convenient and easily accessible	☐	☐	☐	☐

5. Bidirectional referral mechanism between the Hospital Authority (HA) and private primary care physician

5.1 In the last 12 months, have you received a referral from your family doctor?

A referral occurs when a doctor makes an appointment for you to see a different doctor or health professional. For example, this might be for tests, to see a physiotherapist or to see a specialist in hospital. Please do not include referrals within a GP practice (e.g. from a GP to a practice nurse) or referrals from A&E to another part of the hospital when answering this question.

☐ Yes

☐ Yes, I have received a referral in the last 12 months, but the appointment has not been taken yet (proceed to part 6)

☐ No, I have not received a referral in the last 12 months (proceed to part 6)

5.2 How many referrals have you received from your FD in the last 12 months? ________ times

5.3 Which of the following types of medical professionals/ programmes have you been referred to by your FD in the last 12 months? (Multicode)

- ☐ Specialists
- ☐ A& E doctor
- ☐ Allied health professionals (physiotherapists, occupational therapists, dietitians)
- ☐ DHC/DHCE programmes (IDDP, PEP)
- ☐ Others, please specify: _________________

	If you had a referral over the last 12 months: (please ask for every selected option in Q5.3)	**Definitely**	**Mostly**	**Somewhat**	**Not at all**
5.4	I understand the reason for the referral	☐	☐	☐	☐
5.5	I had to request for the referral	☐	☐	☐	☐
5.6	I was given useful information about how to attend	☐	☐	☐	☐
5.7	I needed to seek further medical help for my condition while waiting for the referral appointment	☐	☐	☐	☐
5.8	I was given a choice of where I would be referred	☐	☐	☐	☐
5.9	I was satisfied with the way referral was handled	☐	☐	☐	☐
5.10	Making this referral was necessary for managing my condition	☐	☐	☐	☐

5.11 If answered 'strongly disagree/disagree' for Q5.10:
Why do you think the referral unnecessary?

- ☐ I felt the doctor referred me because they did not have enough time to see me properly themselves
- ☐ I felt the doctor referred me because they were not confident enough to give me a diagnosis
- ☐ I knew my symptoms did not require a referral
- ☐ I think my doctor may have referred me mainly because they knew that I wanted a referral
- ☐ I felt the healthcare professional/service I was referred to was not the right one to help me
- ☐ The medical professional I was referred to said that my referral was
- ☐ Others, please specify: ___________________________

**6.**	**Patient Enablement: After the last 12 months, I feel I am:**	**Greatly improved**	**Slightly improved**	**The same or less**	**N/A**
6.1	able to face my life	☐	☐	☐	☐
6.2	able to understand my illness	☐	☐	☐	☐
6.3	able to live with my illness	☐	☐	☐	☐
6.4	able to maintain good health	☐	☐	☐	☐
6.5	confident about my health	☐	☐	☐	☐

6.6	able to self help	☐	☐	☐	☐

7.	**Overall impression**	**Definitely**	**Mostly**	**Somewhat**	**Not at all**
7.1	I am happy with the coverage amounts provided by the Scheme	☐	☐	☐	☐
7.2	The amount charged for the medications is reasonable (if applicable)	☐	☐	☐	☐
7.3	There were multiple and convenient payment methods	☐	☐	☐	☐
7.4	There was sufficient access to coordinated and multidisciplinary care with the programme	☐	☐	☐	☐
7.5	My medical information is easier to get with the eHealth app	☐	☐	☐	☐
7.6	My medical record confidentiality is improved with the eHealth app	☐	☐	☐	☐
7.7	Using the eHealth app has improved my overall quality of care	☐	☐	☐	☐
7.8	The Scheme makes it easy for me to get care	☐	☐	☐	☐
7.9	This Scheme is able to provide most of my care	☐	☐	☐	☐
7.10	In caring for me, my family doctor considers all factors that affect my health	☐	☐	☐	☐
7.11	My practice coordinates the care I get from multiple places	☐	☐	☐	☐
7.12	My family doctor or practice knows me as a person	☐	☐	☐	☐
7.13	My family doctor and I have been through a lot together	☐	☐	☐	☐
7.14	My family doctor or practice stands up for me	☐	☐	☐	☐
7.15	The care I get takes into account knowledge of my family	☐	☐	☐	☐
7.16	The care I get in this practice is informed by knowledge of my community	☐	☐	☐	☐
7.17	Over time, this practice helps me to meet my goals	☐	☐	☐	☐
7.18	Over time, my practice helps me stay healthy	☐	☐	☐	☐
7.19	How many years have you known this doctor?	☐	☐	☐	☐
7.20	Overall, I am satisfied with the coverage of services provided by the Scheme	☐	☐	☐	☐
7.21	Overall, I am satisfied with the quality of care provided by the Scheme	☐	☐	☐	☐
